# Impact of medication on blood transcriptome reveals off-target regulations of beta-blockers

**DOI:** 10.1371/journal.pone.0266897

**Published:** 2022-04-21

**Authors:** Michael Rode, Kolja Nenoff, Kerstin Wirkner, Katrin Horn, Andrej Teren, Ralf Regenthal, Markus Loeffler, Joachim Thiery, Achim Aigner, Janne Pott, Holger Kirsten, Markus Scholz

**Affiliations:** 1 Institute for Medical Informatics, Statistics and Epidemiology, University of Leipzig, Leipzig, Germany; 2 LIFE Research Center for Civilization Diseases, University of Leipzig, Leipzig, Germany; 3 Department of Cardiology, Angiology and Intensive Care, Klinikum Lippe, Detmold, Germany; 4 Rudolf-Boehm-Institute for Pharmacology and Toxicology, Clinical Pharmacology, University of Leipzig, Leipzig, Germany; 5 Medical Campus Kiel, University Hospital Schleswig-Holstein, Kiel, Germany; Medical University Innsbruck, AUSTRIA

## Abstract

**Background:**

For many drugs, mechanisms of action with regard to desired effects and/or unwanted side effects are only incompletely understood. To investigate possible pleiotropic effects and respective molecular mechanisms, we describe here a catalogue of commonly used drugs and their impact on the blood transcriptome.

**Methods and results:**

From a population-based cohort in Germany (LIFE-Adult), we collected genome-wide gene-expression data in whole blood using in Illumina HT12v4 micro-arrays (n = 3,378; 19,974 gene expression probes per individual). Expression profiles were correlated with the intake of active substances as assessed by participants’ medication. This resulted in a catalogue of fourteen substances that were identified as associated with differential gene expression for a total of 534 genes. As an independent replication cohort, an observational study of patients with suspected or confirmed stable coronary artery disease (CAD) or myocardial infarction (LIFE-Heart, n = 3,008, 19,966 gene expression probes per individual) was employed. Notably, we were able to replicate differential gene expression for three active substances affecting 80 genes in peripheral blood mononuclear cells (carvedilol: 25; prednisolone: 17; timolol: 38). Additionally, using gene ontology enrichment analysis, we demonstrated for timolol a significant enrichment in 23 pathways, 19 of them including either *GPER1* or *PDE4B*. In the case of carvedilol, we showed that, beside genes with well-established association with hypertension (*GPER1*, *PDE4B* and *TNFAIP3*), the drug also affects genes that are only indirectly linked to hypertension due to their effects on artery walls or their role in lipid biosynthesis.

**Conclusions:**

Our developed catalogue of blood gene expressions profiles affected by medication can be used to support both, drug repurposing and the identification of possible off-target effects.

## Introduction

Over the last years, blood-based gene-expression (GE) analyses have been broadly used to identify biomarkers, to detect potential molecular drivers for diseases and to assess molecular phenotypes. This also provided new insights into disease processes, (sub-clinical) disease states, and response to therapy [1].

However, GE is affected by a plethora of factors including the genetic background of a person, the considered tissue, life-style, environmental and disease-related factors [2, 3]. In this regard, the effect of drugs on single GE and defined pathways is still understudied. For pharmacologists the association between drugs and GE is highly relevant as drug based transcriptome analysis could provide new insights into the mechanisms of action of specific drugs. These insights can be used for drug repurposing [4, 5] but also help to identify underlying reasons for off-target effects [6].

Beta-blockers are widely prescribed drugs that cover a wide spectrum of cardiovascular indications. As beta-blockers are inferior to calcium-channel blockers and renin-angiotensin system inhibitors they are considered as second line antihypertensive treatment [7]. They are however effective in long term secondary prevention after myocardial infarction [8] and are also used in the treatment of specific cardiovascular diseases [9]. Beta-blockers are competitive antagonists that block the receptor sites for the endogenous epinephrine and norepinephrine on beta-adrenoceptors that are found on cells of the heart muscles, smooth muscles, arteries, kidneys, airways and other tissues which are part of the sympathetic nervous system. While non-selective beta-blockers block the activation of all types of beta-adrenoceptors, selective beta-blockers only act on designated receptor subtypes (β1 to β3). In this study, we analysed the impact of 83 active substances on whole blood transcriptome. Results were replicated in an independent cohort. To gain deeper insight into the effects of beta blocking agents and their mechanisms of action, we analysed associated genes and pathways in more detail and compared the results with our current knowledge of the drug’s mechanisms.

## Material and methods

### Cohort description

The LIFE-Adult study is a population-based cohort study of 10,000 participants from Leipzig, a city in Germany [10]. Most of the participants are aged between 40 and 79, with a small subgroup of 400 participants being between 18 and 39. The study population is of central European descent and the main study goal is to investigate prevalence, genetic predisposition and the role of lifestyle-related factors (such as smoking habits, alcohol consumption, dietary patterns and physical activity) on major civilization diseases including subclinical signs. Initial data collection was performed between 2011 and 2014.

LIFE-Heart is an observational study of patients collected at the Heart Center of Leipzig, Germany. A total of 6,994 patients were recruited with suspected or confirmed stable coronary artery disease (CAD) or myocardial infarction. The study design and a detailed description of patients can be found elsewhere [11]. Initial data collection was performed between 2006 and 2014. For the present analysis, we excluded patients with acute myocardial infarction since the acute situation may have a profound impact on gene expression profiles.

Baseline characteristics for the cohorts are provided in Table 1.

**Table 1:** Study characteristics of LIFE-Adult and LIFE-Heart.

Study characteristics				
*Parameter*	*LIFE-Adult (n = 3*,*378)*		*LIFE-Heart (n = 2*,*978)*	
	*With medication*	*Without medication*	*With medication*	*Without medication*
Men / Women	1323 / 1419	422 / 214	1849 / 1003	99 / 27
Age (years)	60.7 ± 12.1	51.1 ± 11.6	63.2 ± 10.8	53.9 ± 10.4
Non Smoker / Smoker	2061 / 485	432 / 183	2344 / 508	90 / 36
BMI (kg/m^2^)	27.9 ± 4.8	26.1 ± 3.8	29.8 ± 5.0	27.3 ± 3.8
Lymphocytes in %	29.8 ± 7.9	32.2 ± 7.6	25.4 ± 7.7	28.1 ± 7.3
Monocytes in %	8.2 ± 2.1	8.3 ± 2.0	8.7 ± 2.3	8.6 ± 2.6
Average number of active substances per individual	4.1 (median = 3, IQR = 2 to 6)	0	5.9 (median = 5, IQR = 3 to 8)	0

For the continuous parameters, the arithmetic mean and SD is given. Additionally, average numbers of substances are given as median and interquartile range (IQR).

Both studies meet the ethical standards of the Declaration of Helsinki and were approved by the Ethics Committee of the Medical Faculty of the University Leipzig, Germany (LIFE-Adult: Reg. No 263-2009-14122009; LIFE-Heart: Reg. No. 276–2005). Written informed consent including agreement with molecular-genetic analyses was obtained from all participants.

### Gene expression analysis

RNA was available from whole blood of n = 3,526 LIFE-Adult participants. Raw gene-expression data were measured by Illumina HumanHT-12 v4 Expression BeadChip. A total of 47,231 expression probes were successfully measured in all samples using Illumina GenomeStudio. We further processed these data within R 2.13.1 / Bioconductor. Transcripts not sufficiently expressed according to Illumina’s internal cut-off as implemented in Bioconductor package ‘lumi’ (detection p-value≤ 0.05) in at least 5% of all samples were not further considered in the analysis. Expression values were quantile normalised and log2-transformed [12]. Furthermore, we defined for each individual a combined quantitative measure combining quality control features available for HT-12 v4 (i.e. perfect-match and miss-match control probes, control probes present at different concentrations, mean of negative control probes, mean of house-keeping genes, Euclidean distances of expression values, number of expressed genes, mean signal strength of biotin-control-probes, S1 Fig). We calculated the Mahalanobis-distance between all individuals and an artificial individual showing average values for these quality control features (S2 Fig). Samples had to be within 4 x interquartile range (IQR) from the median [13] of this distance. Transcript levels were adjusted for the known batch Sentrix barcode (i.e. expression chip-ID) using an empirical Bayes method as described [14]. The empirical Bayes method required that at least two individuals for each batch are provided. This excluded two individuals. Success of adjustment was checked using ANOVA for both, the Sentrix barcode as well as the processing batch (in a processing batch, several expression chips were jointly processed, in consequence, within a processing-batch, several Sentrix barcodes are nested, S3 Fig). Finally, we controlled for the Euclidean distance between all samples and an artificial sample defined as the average of samples (after removing 10% samples farthest away from the average of all samples). We found no individual with a distance larger than median + 4 x IQR. The final sample size was n = 3,378. As previously described [15], we filtered gene expression probes for sufficiently good mapping leaving a final number of 19,974 valid gene expression probes corresponding to 13,693 unique genes. The described pre-processing pipeline has been published as R-package HT12ProcessoR on GitHub [16]. The pre-processing method corresponds to the method named “noBg_log_quantile” and was found to be one of the two best-performing methods regarding optimal bias and variance performance [12] (S4 Fig)o,

In LIFE-Heart, RNA from peripheral blood mononuclear cells (PBMC) was used (n = 3,008). Gene expression data was processed and filtered as described above resulting in final sample-size n = 2,978 and 19,966 valid gene expression probes mapping to 13,687 unique genes.

### Drug assessment

Participants of LIFE-Adult were asked to provide all packages of medicaments taken in the last 7 days to the study centre. Packages were recorded electronically. In LIFE-Heart, patient medical records were evaluated to determine the current medication. For both cohorts no information about previous medication is available.

Results were classified based on the German anatomic therapeutic chemical (ATC) classification [17], which is the German translation of the ATC/DDD Index published by the WHO Collaborating Centre for Drug Statistics Methodology, Oslo. The ATC-Classification is a five level system dividing substances into different groups according to the organ or organ system that they affect and their pharmacological and therapeutic properties. The active substance is named in the lowest level (level five). Based on the ATC codes provided for each study participant and the level 5 information available, we compiled a list of active substances for each individual. As shown in Table 1 we had in both cohorts participants taking no medication, which were used as control group.

### Statistical analysis

Statistical analysis was performed using the statistical software package R 3.6.0. Gene expression analysis was performed using the R-packages lumi 2.3.8 [18] and limma 3.42.2 [19].

#### Polymedication

Dose of medication was not available; therefore, we considered medication of active substances as binary traits. To account for the effects of polymedication in our analysis, we aimed to adjust for the drugs with the largest impact on gene-expression in our models. To define these, we first performed multivariate linear regression analysis of gene-expressions estimating the impact of fifteen substances that were used by more than five percent of our LIFE-Adult subjects. In this analysis, we adjusted for sex, age, lymphocytes, monocytes, smoking status, and log transformed Body-Mass-Index (BMI) and tested one of the fifteen active substances at a time. For our gene expression adjustment model we selected active substances that caused significantly differential gene expression (5% FDR per substance).

We tested collinearity between the substances in the adjustment model using variance inflation factor (VIF), confirming that there is no multi-collinearity problem between the resulting variables used for adjustment.

#### Multivariate differential gene expression analysis

Next, we used the adjustment model defined above to analyse the effects of the single active substances on gene expression. We first tested for an extreme pairwise odds ratio (OR < 0.125 or OR > 8) between the active substance considered and the drugs in the adjustment model. If such an extreme pairwise OR was detected, the respective drug of the adjustment model was dropped to avoid collinearity issues. A total of 83 active substances were analysed (each of them used by at least 20 LIFE-Adult participants) (S1 Table: Active substances analysed). P-values for each active substance were estimated based on moderated t-statistics [20] and adjusted according to Benjamini and Hochberg’s method to control the false discovery rate (FDR) [21]. Significant probes identified were mapped to the respective genes. A gene is considered significantly differentially expressed, when at least one probe mapped to this gene was significantly differentially expressed (q ≤ 0.05).

**Fig 1:**
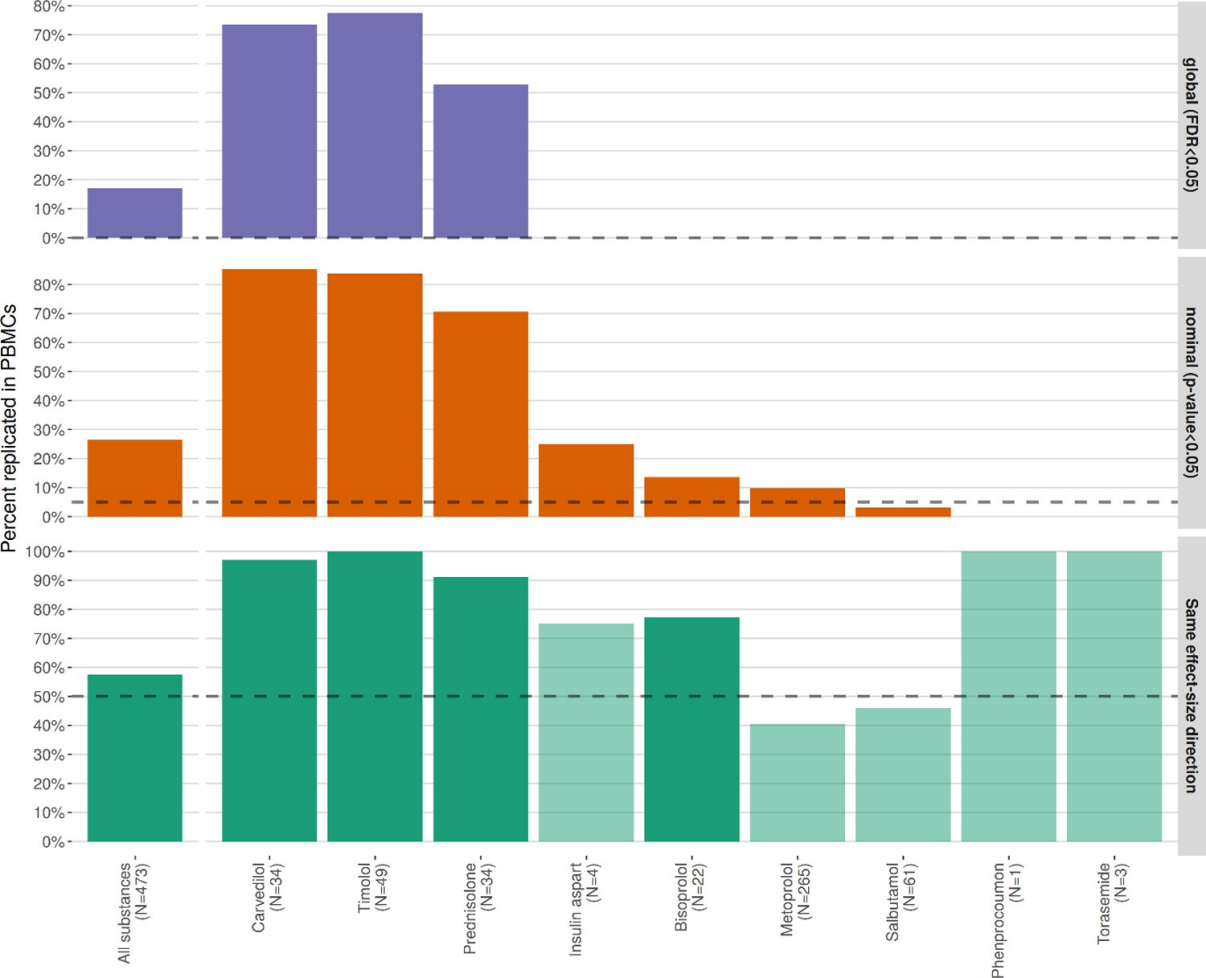
Replication of LIFE-Adult results in LIFE-Heart. Top graph shows results of hierarchical multiple testing correction, which we selected as replication criteria. For information purposes, we include results for nominal significance (graph in the middle) and sign-test (lower graph). Results show strong transferability of results for whole blood to PBMC for carvedilol, timolol and prednisolone.

We aimed at replicating the identified associations in the LIFE-Heart cohort using the same adjustment model for all probes that were significant in LIFE-Adult. For replication, we applied hierarchical multiple testing correction. First, we adjusted the p-values for each substance by Benjamini and Hochberg’s method [21] resulting in q-values per substance. Then, for each substance we selected the lowest q-value for further calculation. In the next step, these lowest q-values per substance were taken and further adjusted for multiple testing of substances according to Benjamini and Bogomolov’s method for multiple testing in families of hypotheses [22]. The result for a gene was considered replicated when at least one probe that mapped to a gene was significantly differentially expressed in LIFE-Heart and showed the same effect direction for the same drug. In addition we performed a sensitivity analysis, by comparing the results with those obtained from a sign-test and nominal significance (Fig 1).

For the replicated substances and genes, we performed a pathway enrichment analysis considering all analysed genes as background. Here, we used ontologies KEGG, GO, DOSE and Reactome [23–26] and considered an FDR value per substance of 0.05 as cut-off. The complete analysis workflow is shown in S5 Fig.

## Results

### Polymedication

**Fig 2:**
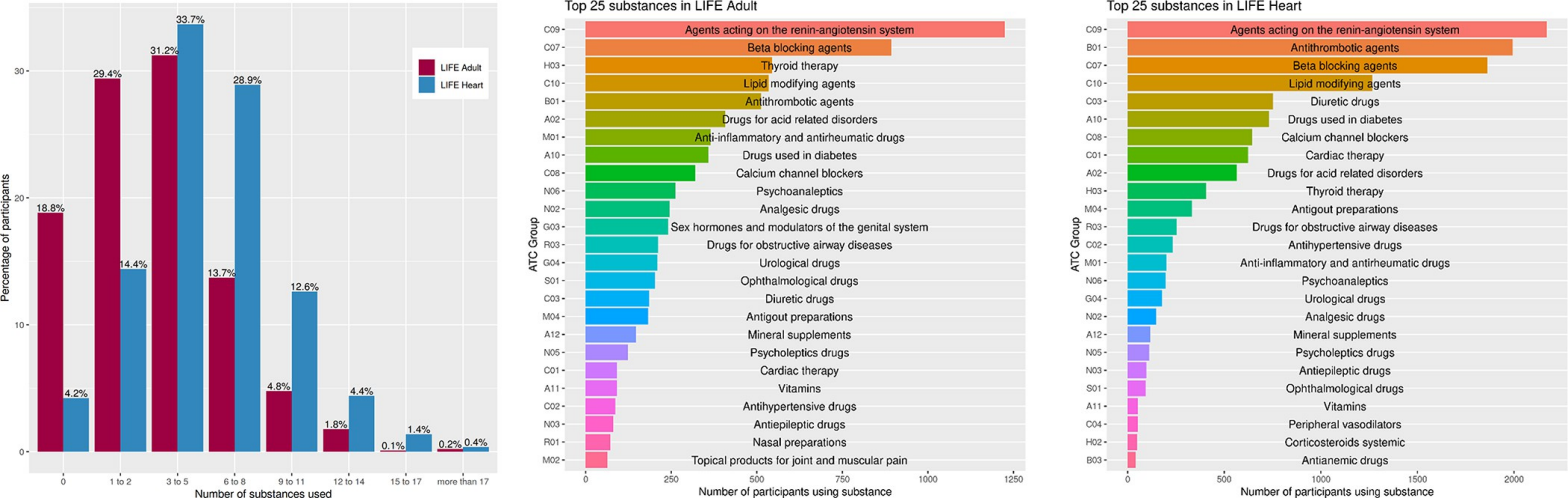
Polymedication of LIFE-Adult and LIFE-Heart participants and most common substances. Top: Polymedication of LIFE-Adult and LIFE-Heart participants, shown by number of active substances consumed. Participants taking no medication were used as control group. Bottom: Most common substances used in both cohorts.

The majority of study participants took more than one active substance (Fig 2). Most participants hereby took medication affecting the cardiovascular system. In total, we identified 745 drugs with 587 different active substances in our LIFE-Adult discovery cohort (LIFE-Heart: 568 drugs; 512 active substances). From the 587 active substances taken by LIFE-Adult participants, we considered 15 substances as potential covariates to adjust for polymedication. Among these, eight substances in LIFE-Adult caused significantly (FDR ≤ 0.05) differentially expressed gene expression probes (Table 2). Thus, we adjusted for a total of eight substances in our final model for gene expression analyses in conjunction with sex, age, lymphocytes, monocytes, smoking status and log transformed Body-Mass-Index (BMI).

**Table 2:** Active substances showing significant effects on gene expression levels in LIFE-Adult and hence adjusted for in gene expression analysis.

	*LIFE Adult*		*LIFE Heart*	
	*# (%) of study participants taking substance*	*# of significant probes (fdr≤0*.*05)*	*# (%) of study participants taking substance*	*# of significant probes (fdr≤0*.*05)*
Acetylsalicylic acid	436 (15.9%)	9	1,681 (58.9%)	1
Allopurinol	176 (6.4%)	387	330 (11.6%)	6
Bisoprolol	439 (16.0%)	1,529	805 (28.3%)	0
Hydrochlorothiazide	179 (6.5%)	5	716 (25.1%)	11
Metformin	274 (8.1%)	210	396 (13.2%)	14
Metroprolol	297 (8.8%)	3,597	740 (25.9%)	1
Simvastatin	454 (13.4%)	558	1022 (35.9%)	5
Valsartan	211 (6.2%)	8	294 (10.3%)	0

These substances were used to account for polymedication in the multivariate analysis models for both studies. In LIFE-Adult n = 636 (LIFE-Heart n = 126) participants did not take medication.

### Differential gene expression analysis

Using the polymedication based adjustment model, we identified fourteen (of initially analysed 83 substances taken by 20 or more LIFE-Adult participants) active substances that significantly (q ≤ 0.05) affected the expression of a total of 544 probes matching 534 genes in LIFE-Adult (S2 Table: Summary of significant substances). The ratio between up and down regulation was balanced (down regulation: n = 248; up regulation n = 286). Effect sizes varied between β = -0.75, q = 2.36x10^-2^ (effect of Propranolol on *GZMB*) and β = 0.45, q = 7.84x10^-6^ (effect of Carvedilol on *ADRB2*). The number of genes affected by an active substance varied between one gene for phenprocoumon and 265 genes being affected by metoprolol. The fourteen substances included two non-selective (carvedilol, propranolol) and two selective (bisoprolol, metoprolol) beta-blockers which are primarily used to treat hypertension and cardiovascular diseases. A complete list of the analysed active substances and affected genes is provided in S3 Table (statistics for each significant probe / substance combination). With respect to the number of genes affected by a single active substance our study shows that beta blockers affect the highest number of genes (376 of a total of 534 genes shown to be affected by drugs from this class in our study), followed by bronchodilators (59 genes), estrogen (46 genes) and corticosteroids (33 genes).

Matching the significant probes with available LIFE-Heart probes resulted in 473 probes and 465 genes eligible for replication. This excludes 71 probes were the active substance was ethinylestradiol, levonorgestrel, propranolol, thiazide or vildagliptin which were not recorded in LIFE-Heart. Thus, a total of 473 probes associated with nine active substances were tested in LIFE-Heart. The aggregated replication results are provided in Table 3.

**Table 3:** Substances that cause differential gene expression in LIFE-Adult and their replication in LIFE-Heart.

Substance	number sign. probes LIFE-Adult	thereof:		Minimum q-value	number probes available in LIFE-Heart	number sign. probes LIFE-Heart	thereof:		Minimum q-value
		*up-regulated*	*down-regulated*				*up-regulated*	*down-regulated*	
Bisoprolol	22	6	16	5.03x10^-04^	22	0	0	0	2.56x10^-01^
Carvedilol	34	30	4	1.57x10^-10^	34	25	22	3	1.50x10^-12^
Ethinyl estradiol	47	33	14	2.30x10^-03^	0	n/a	n/a	n/a	n/a
Insulin aspart	4	2	2	4.80x10^-02^	4	0	0	0	4.41x10^-01^
Levonorgestrel	8	7	1	2.75x10^-02^	0	n/a	n/a	n/a	n/a
Metoprolol	265	107	158	2.05x10^-04^	265	0	0	0	4.90x10^-01^
Phenprocoumon	1	1	0	1.88x10^-02^	1	0	0	0	9.14x10^-01^
Prednisolone	34	25	9	1.64x10^-05^	34	17	14	3	3.33x10^-07^
Propranolol	11	6	5	7.14x10^-04^	0	n/a	n/a	n/a	n/a
Salbutamol	61	41	20	1.54x10^-02^	61	0	0	0	8.91x10^-01^
Thiazide	1	1	0	2.74x10^-02^	0	n/a	n/a	n/a	n/a
Timolol	49	27	22	9.14x10^-09^	49	38	20	18	1.73x10^-04^
Torasemide	3	1	2	1.87x10^-02^	3	0	0	0	3.06x10^-01^
Vildagliptin	4	4	0	1.24x10^-02^	0	n/a	n/a	n/a	n/a

Not all probes significant in LIFE-Adult were available in LIFE-Heart. Minimum q-value refers to the probe associated with a substance with lowest q-value.

**Fig 3:**
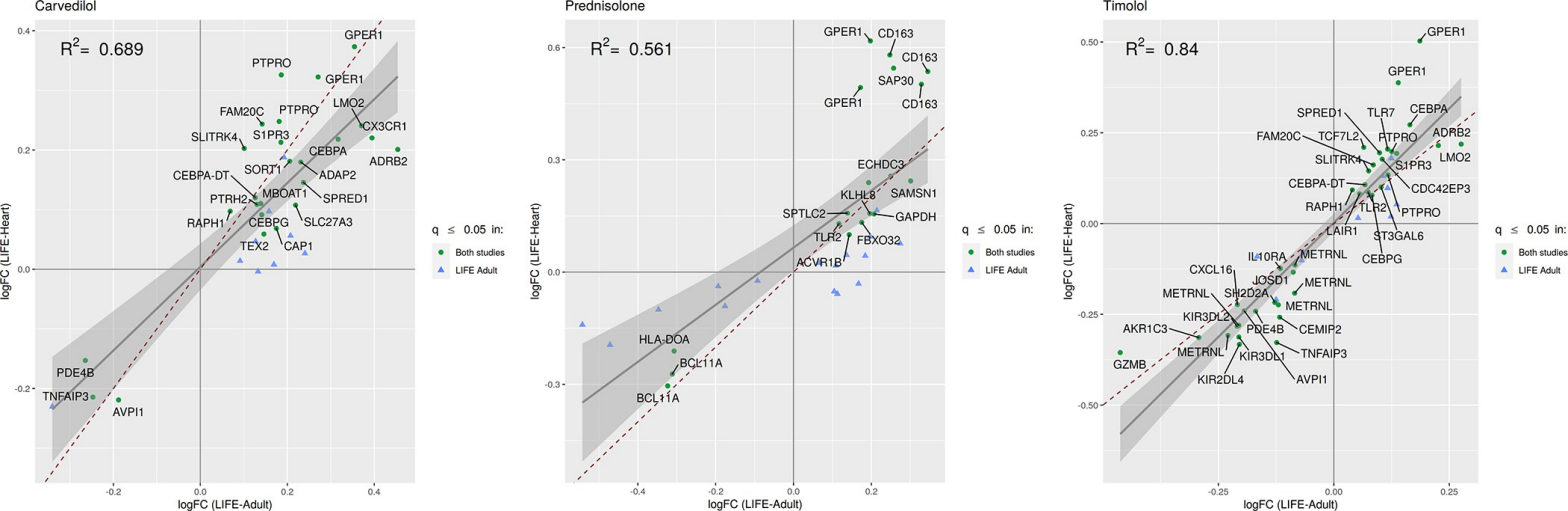
Differential gene expression caused by carvedilol, prednisolone and Timolol. Original results as obtained from LIFE-Adult and successfully replicated in LIFE-Heart. Genes may be captured on multiple probes and are then shown multiple times. All replicated genes show the same effect direction.

**Fig 4:**
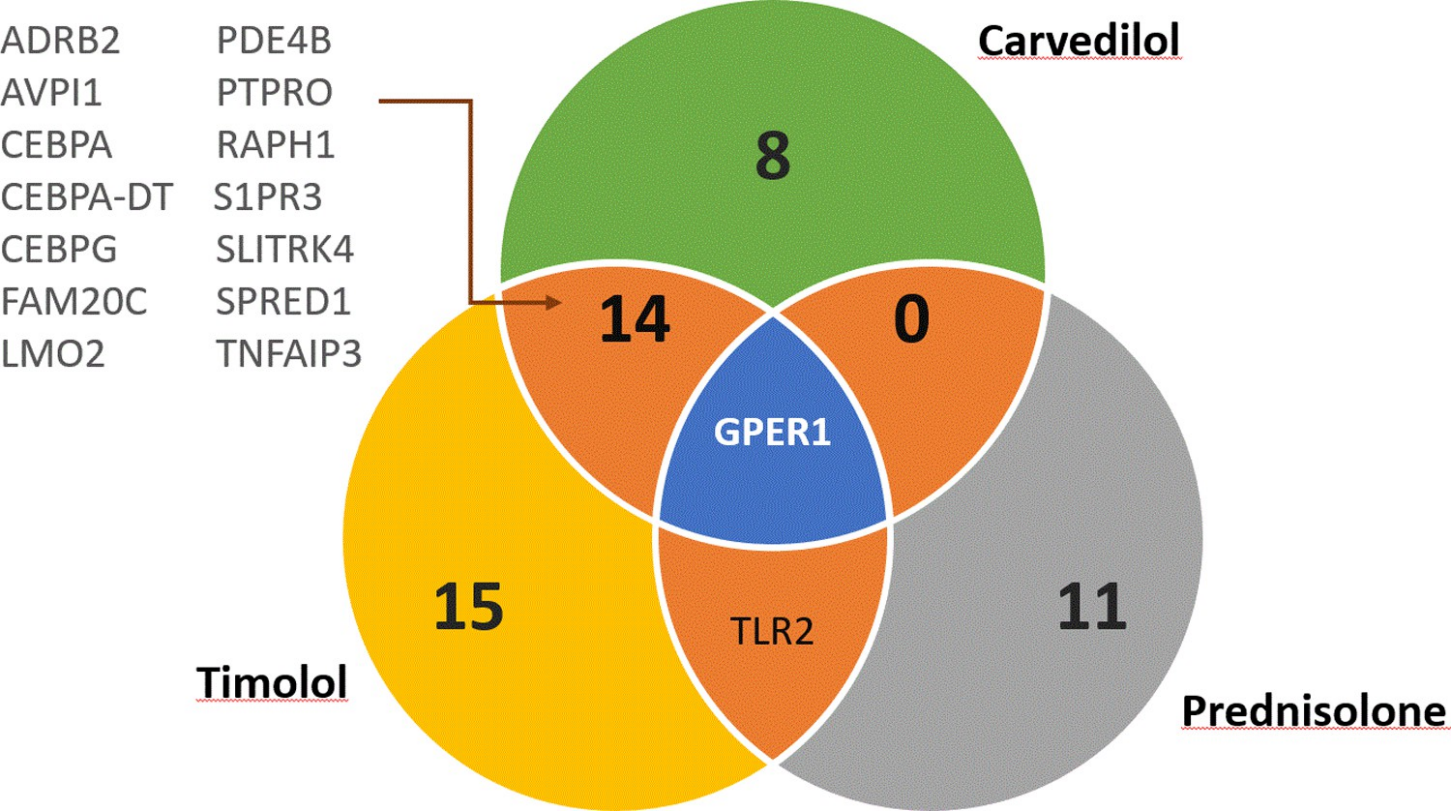
Genes overexpressed by more than one substance. Analysis shows high overlap between timolol and carvedilol.

**Fig 5:**
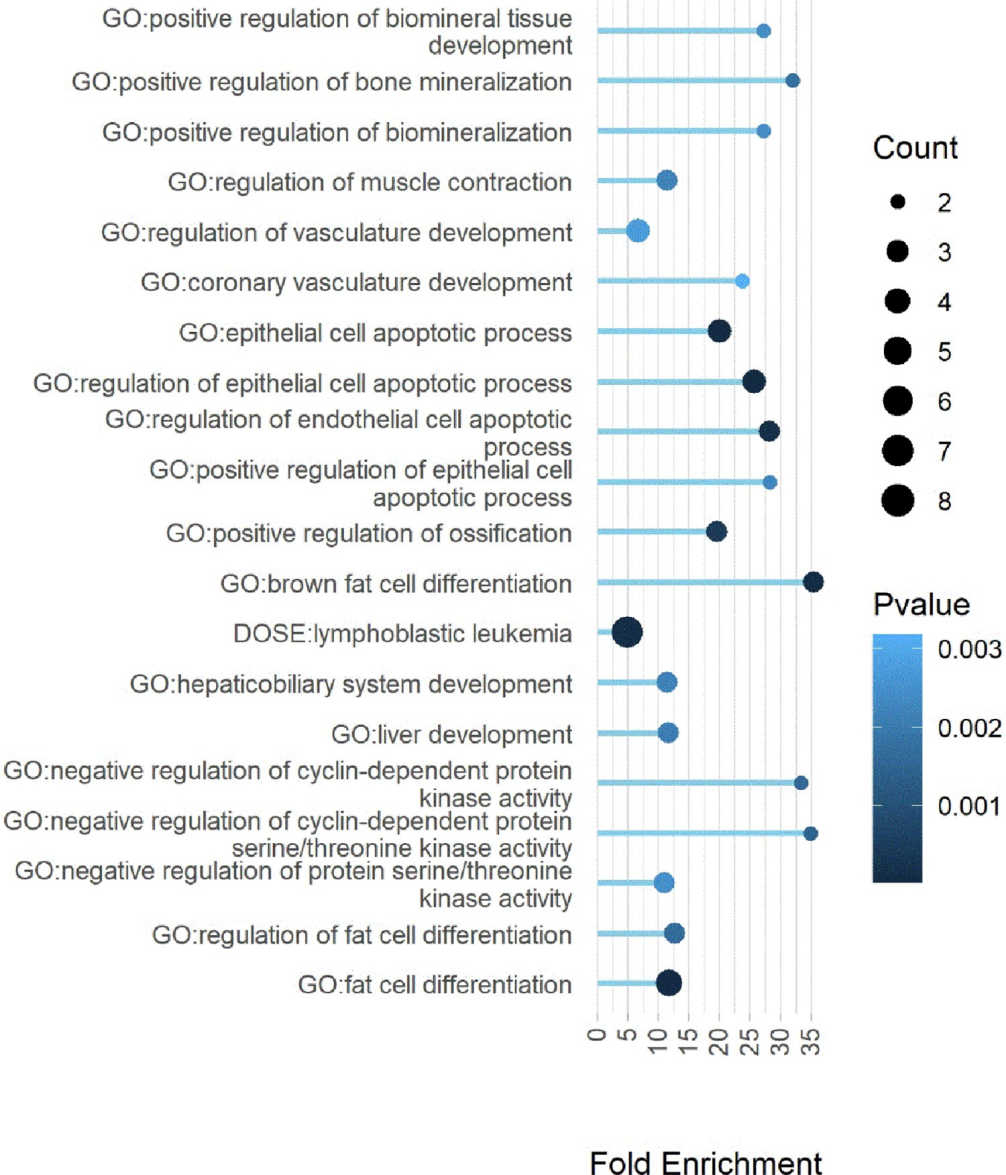
Pathways with significant enrichment in LIFE-Adult and LIFE-Heart (FDR < 5%). If two pathways are enriched due to the identical set of replicated genes, only the pathway with the higher enrichment (i.e. higher Odds ratio) is shown here. All significantly enriched pathways are reported in S5 Table. Differentially expressed genes per pathway are shown in S6 Fig.

For three of the nine active substances (carvedilol, prednisolone and timolol), we were able to also show differential gene expression in PBMC in LIFE-Heart (Fig 3). More specifically, we could replicate 25 of the originally identified 34 probes for carvedilol, 17 of the originally identified 33 probes for prednisolone and 38 of originally 49 probes in the case of timolol. For all three substances the differential gene expression of all replicated genes had the same direction in both cohorts. Between carvedilol and timolol there was an overlap of 15 genes (Fig 4), causing an upregulation, among others, of *GPER1*, *PDE4B* and *TNFAIP3*, which are genes directly associated with hypertension [27–29]. The full list of replicated genes and their effect strength and -direction is provided in S4 Table. For all three substances we also identified a total of 120 significantly enriched pathways of replicated genes. The majority of the enriched pathways were caused by the beta blockers timolol (69 enriched pathways) and carvedilol (48 enriched pathways). For Prednisolone we identified three enriched pathways. Between timolol and carvedilol there was an overlap of 20 enriched pathways (Fig 5, S6 Fig, S5 Table). Of the 20 pathways, 15 included either GPER1 or TNFAIP3.

## Discussion

At the current state of research, the effects of drugs on defined gene expression profiles is understudied. We here performed the first population based transcriptome-wide association analysis of 83 drugs on gene expression in two independent cohorts. We discovered 534 genes affected by 14 substances in whole blood in our cohort of 3,378 subjects of LIFE-Adult. Notably, we were able to replicate differential gene expression for three drugs affecting 80 genes in peripheral blood mononuclear cells (carvedilol: 25; prednisolone: 17; timolol: 38).

Replication in LIFE-Heart shows the transferability of our results from whole blood to PBMC on gene as well as on pathway level. As PBMC represents only a subset of the cells available in whole blood the replication is even more notable. This was true for the two beta-blockers, carvedilol, timolol, as well as for prednisolone. Using more relaxed criteria for replication (nominal significance and sign-test) also led to results for bisoprolol that could be replicated. While timolol is used as long term medication in form of eye drops to treat glaucoma and decrease intraocular pressure, the results confirm gene expression due to systemic concentrations after local application [30] as timolol avoids to a large extend the first pass metabolism and about 80% of the drug are systemically absorbed [31]. This is in line with the large effect of timolol, which we observed at both, gene- and pathway level.

We were not able to compare our results with other studies, because population based gene expression studies for substances have not been published yet. The only study that had analysed the effect of carvedilol on gene expression was for heart tissue in mice [32]. There was no overlap between the genes found in this study and the genes identified by us. To better understand if our results provide potential insights into possible off-target-effects, we therefore searched the literature for the genes that were affected in both cohorts by carvedilol, a commonly used beta- and alpha-blocker that causes vasodilation. Only three of the 23 unique genes replicated, have been directly associated with hypertension (*GPER1*, *PDE4B* and *TNFAIP3*) so far [27–29]. All three genes were also differentially expressed by timolol. Further eight genes are associated with having effects on artery walls (*LMO2* [33], *ADRB2* [34], *SPRED1* [35], *CX3CR1* [36], *ADAP2* [37], *PTPRO* [38], *RAPH1* [39] and *CEBPG* [40, 41]) and are therefore indirectly linked to blood pressure. Another six genes (*S1PR3* [42], *MBOAT1* [43], *FAM20C* [44], *CAP1* [45], *SORT1* [46] and *SLC27A3* [47]) are involved in lipid biosynthesis and can therefore affect blood pressure indirectly via influencing atherosclerosis. For the remaining six genes, no studies exist so far linking them to hypertension. These genes are *CEBPA*, *CEBPA-DT*, *SLITRK4*, *TEX2*, *AVPI1* and *PTRH2*. As beta blockers affect the whole sympathetic nervous system, effects other than on the cardiovascular system are not surprising. Further research is needed to clarify if the differential expression observed for these genes may lead to unwanted side-effects of carvedilol in regards to the cardiovascular system or if the genes indeed are involved in cardiovascular pathomechanisms. Regarding the 17 genes linked directly or indirectly to hypertension, we showed that carvedilol delivers cardioprotective effects on multiple levels. They either affect blood pressure directly, support the repair response to acute cardiac damage or decrease the risk of plaque formation. Also plausible pathway enrichments were found, e.g. negative regulation of interleukin-1 production and brown fat cell differentiation, which are linked to lower blood pressure [48] and better cardio-metabolic health [49].

Limitations: Both analysed studies are cross-sectional, i.e. we could not compare gene-expression prior and after start of medication. We cannot exclude that effects of active substances on blood gene-expressions are at least in parts caused by the underlying disease conditions. Replication of the LIFE-Adult results in LIFE-Heart may be restricted by the different tissues analysed (whole blood vs. PBMC). Differences between LIFE-Adult and LIFE-Heart in the medication anamnesis as well as missing temporal aspects for medication history may have affected the results of differential gene-expression. In LIFE-Adult the medicaments taken in the last 7 days were reported, while in LIFE-Heart only the current medication (based on medical records) was available. No information about previous medication or recent changes in medication was available in both studies. As we analysed the effect on the level of active substances, the medication specific resorption may have affected the results. We also have to acknowledge that case numbers differ largely between different types of medication resulting in largely different power to detect associations. Thus, number of identified genes is not a measure of the total impact of the respective drug on gene-expression.

In conclusion, this is the first study that provides insights on how active substances may affect blood gene-expression. Several novel associations contribute to the understanding of pleiotropic effects and mechanisms of actions of the investigated substances.

## Supporting information

S1 Table Active substances analysed for differential gene expression in in LIFE Adult. Number of users in LIFE Adult and LIFE Heart.
(XLSX)

S2 Table Active substances in LIFE-Adult causing differential gene expression. Active substances causing significant changes in genes expression after adjustment by multivariate model in LIFE Adult. In some cases, multiple probe sets map to the same gene. The total number of unique genes with differential expression is 442. Some genes were effected by more than one active substance.
(XLSX)

S3 Table Active substances and differentially expressed genes. Not all probes identified as significant in LIFE-Adulte were available in LIFE-Heart. Some probes were not linked to a gene.
(XLSX)

S4 Table Genes with differential expression caused by Carvedilol, Prednisolone and Timolol. We present results of LIFE-Adult and their replication in LIFE-Heart. Genes captured on multiple probes and are shown for each probe. Data sorted by LIFE-Adult q-value.
(XLSX)

S5 Table Enrichment analysis for genes that were significant in LIFE-Adult and LIFE-Heart. For enrichment analysis we used a p-value of 0.05 as cut of point. For all three substances significant pathways were identified. Timolol and Carvedilol show overlap of 20 pathways.
(XLSX)

S1 Fig Distribution of attributes for filtering technically failed chips. Distribution of attributes for filtering technically failed chips for LIFE-Heart.
(GIF)

S2 Fig Distribution Mahalanobis-distance. Mahalanobis distance for LIFE-Heart.
(GIF)

S3 Fig ANOVA test results. ANOVA test results for Sentrix and fileset-id before and after Combat for LIFE-Heart.
(GIF)

S4 Fig Preprocessed expression values (nomralized and transformed). Exemplarily shown (for the top-probes of Carvedilol, Prednisolone und Timolol), distribution of pre-processed, i.e. normalized and transformed data. Dashed-line represents normal distribution. Coloured area shows actual data after transformation and normalization.
(GIF)

S5 Fig Study design schematic for discovery and validation of genes. Despriction of processing steps performed.
(GIF)

S6 Fig Differentially expressed genes per pathway. Differentially expressed genes and associated pathways.
(GIF)
