# Supplementary figures and images for: Impact of medication on blood transcriptome reveals off-target regulations of beta-blockers
Source: PLoS One. 2022 Apr 21;17(4):e0266897. doi: 10.1371/journal.pone.0266897 (PMC9022833; doi:10.1371/journal.pone.0266897)

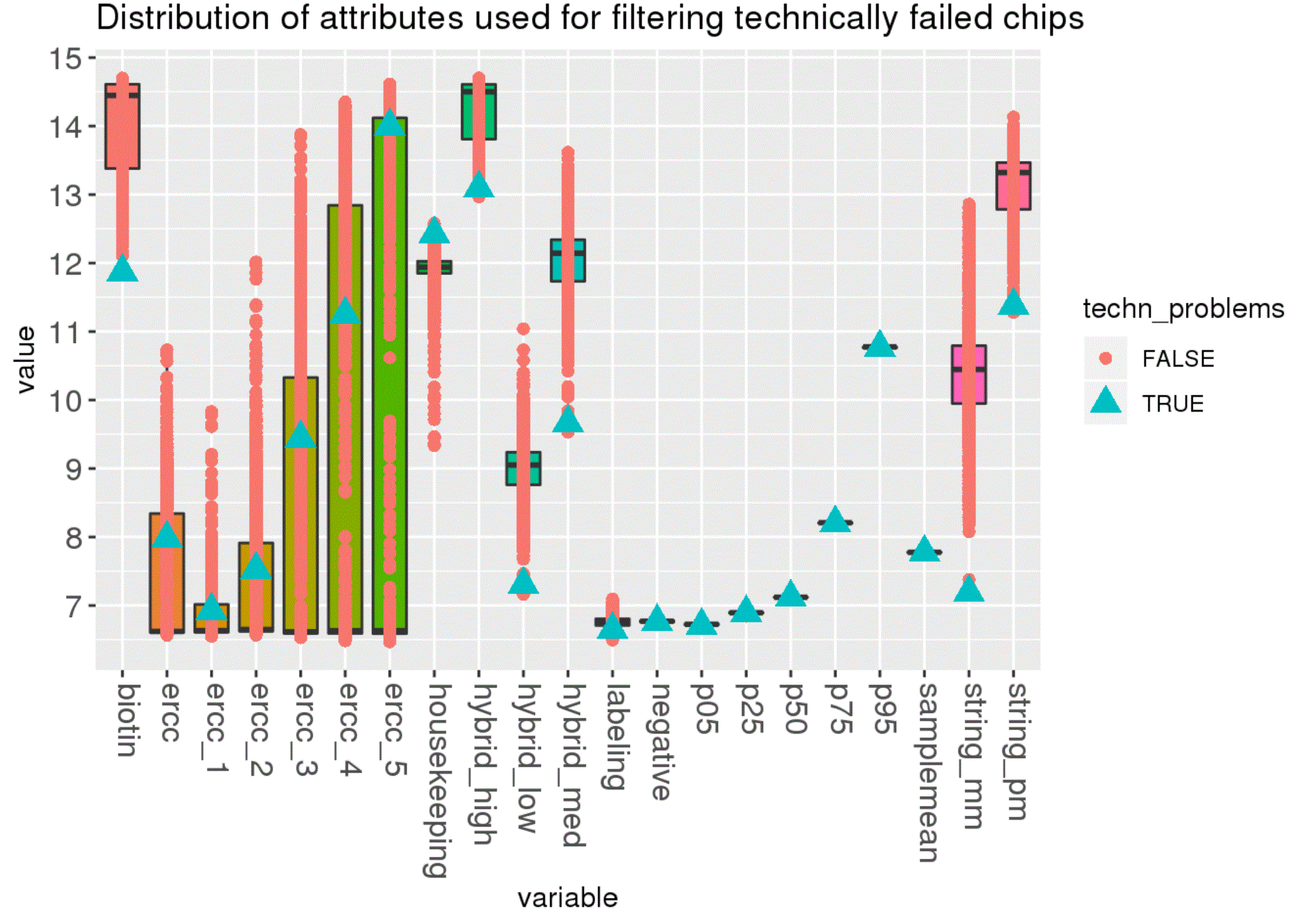

Supplement: S1 Fig — Distribution of attributes for filtering technically failed chips for LIFE-Heart. (GIF) [file pone.0266897.s006.GIF]

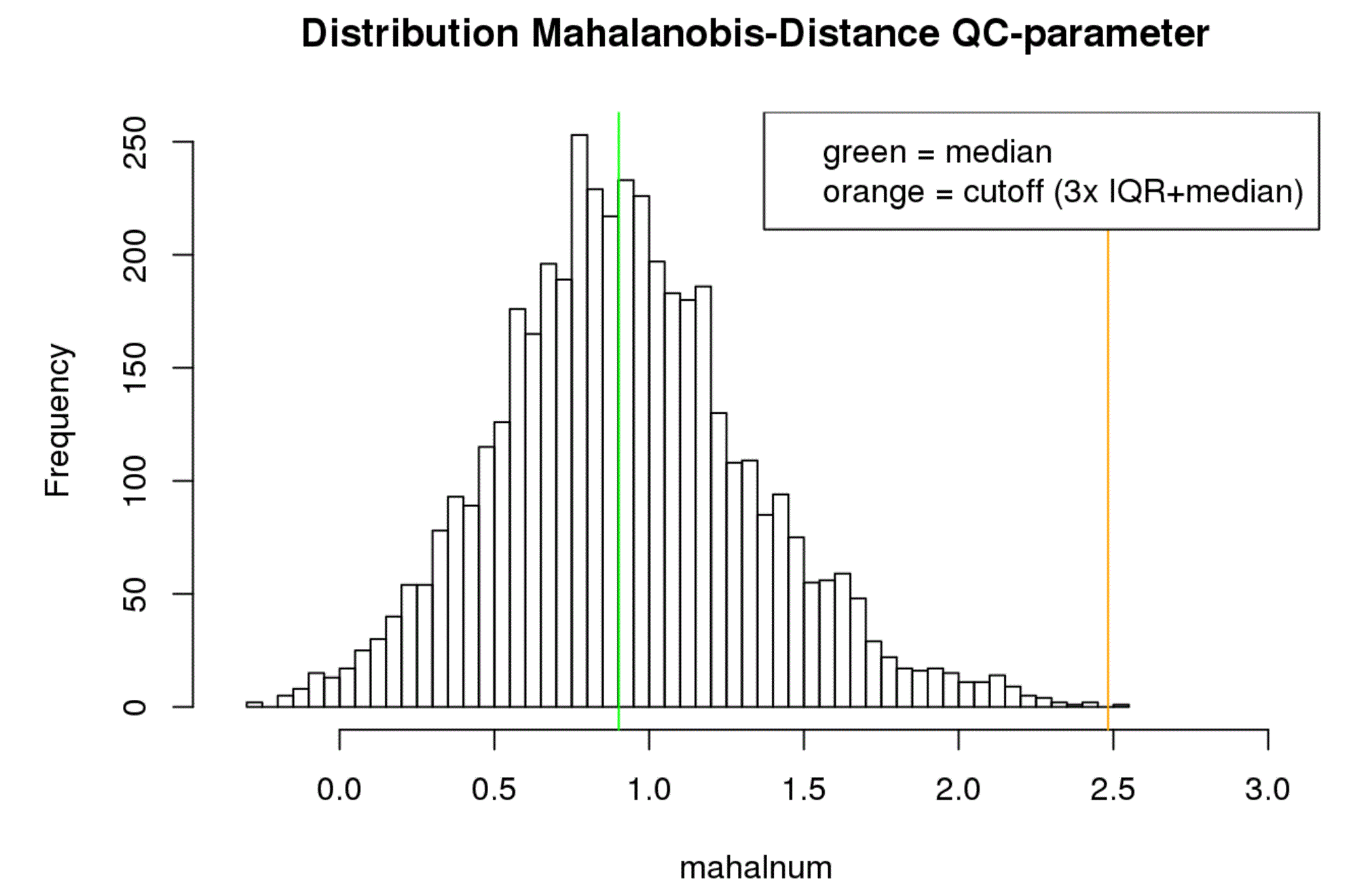

Supplement: S2 Fig — Mahalanobis distance for LIFE-Heart. (GIF) [file pone.0266897.s007.GIF]

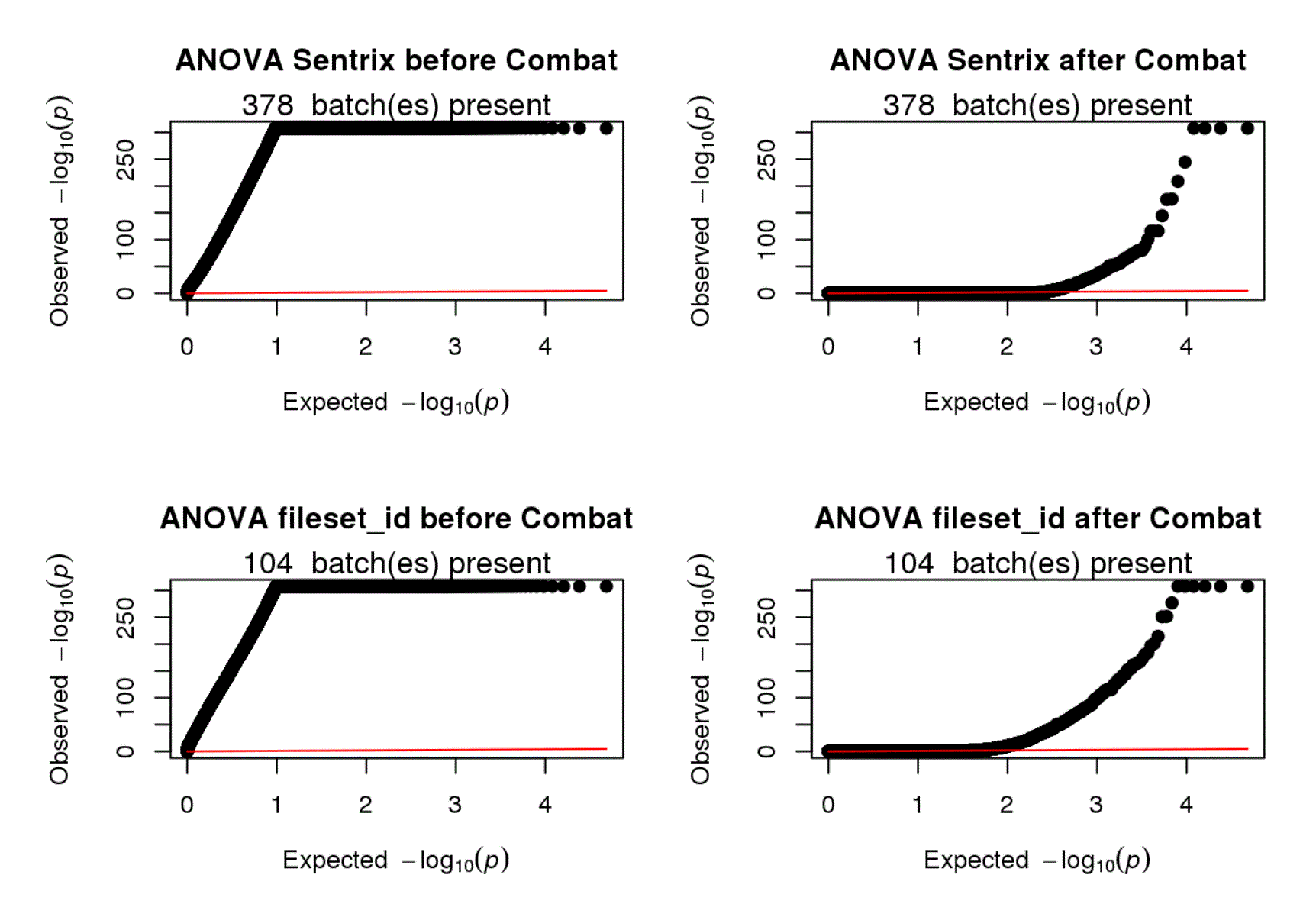

Supplement: S3 Fig — ANOVA test results for Sentrix and fileset-id before and after Combat for LIFE-Heart. (GIF) [file pone.0266897.s008.GIF]

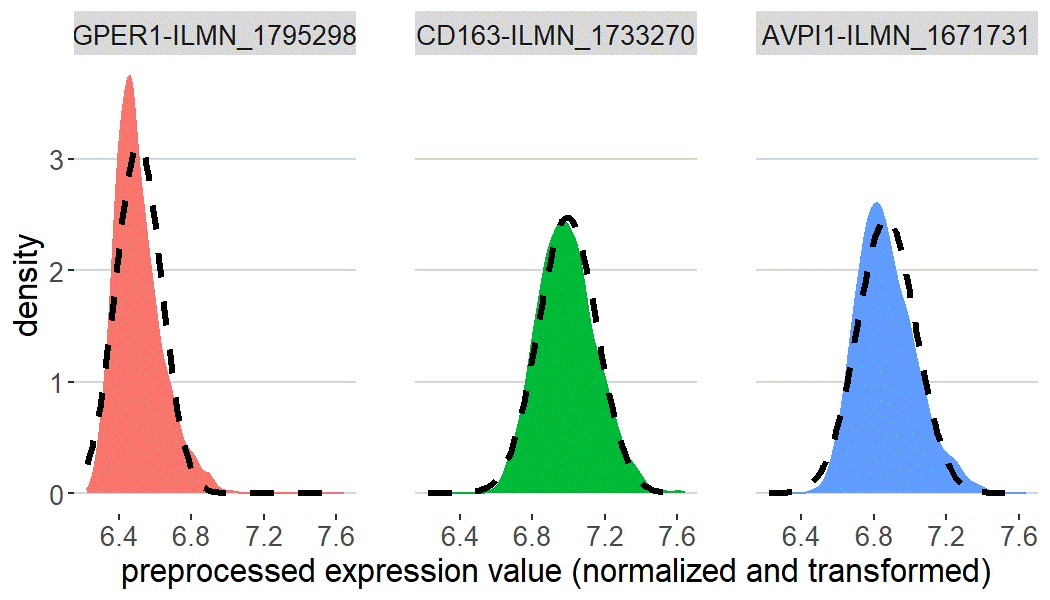

Supplement: S4 Fig — Exemplarily shown (for the top-probes of Carvedilol, Prednisolone und Timolol), distribution of pre-processed, i.e. normalized and transformed data. Dashed-line represents normal distribution. Coloured area shows actual data after transformation and normalization. (GIF) [file pone.0266897.s009.gif]

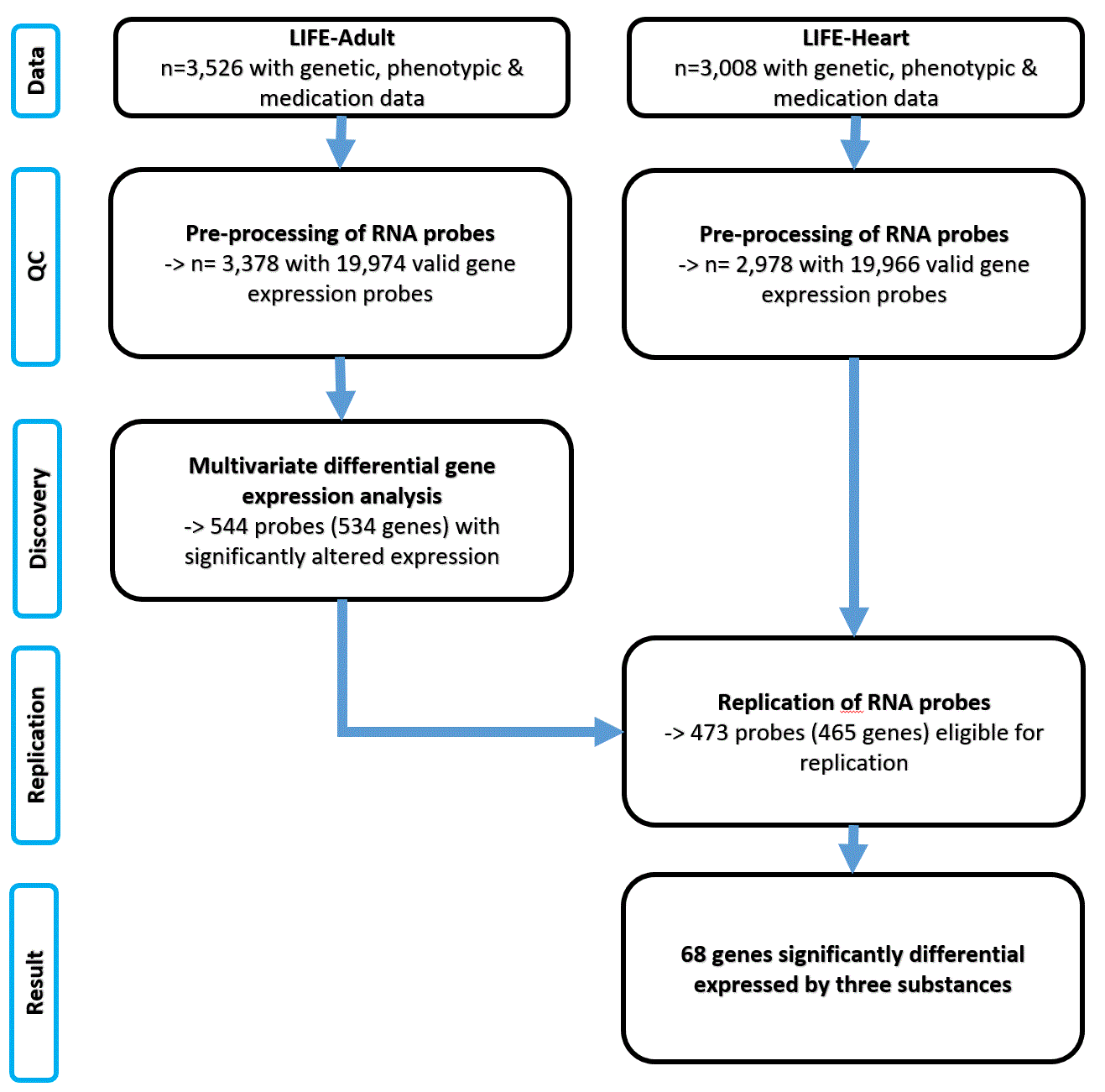

Supplement: S5 Fig — Despriction of processing steps performed. (GIF) [file pone.0266897.s010.GIF]

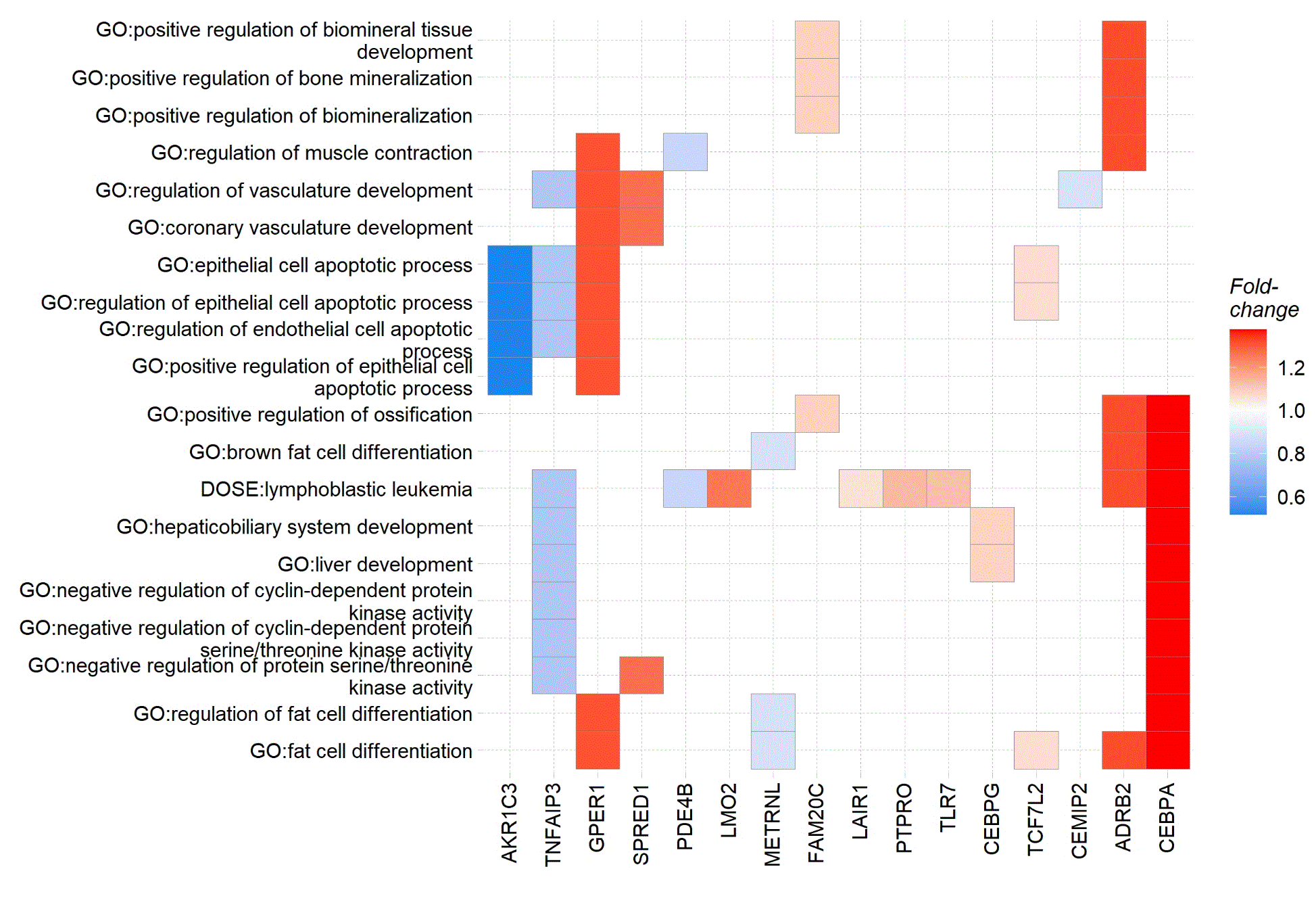

Supplement: S6 Fig — Differentially expressed genes and associated pathways. (GIF) [file pone.0266897.s011.GIF]
